# Research on Personalized Book Recommendation Based on Improved Similarity Calculation and Data Filling Collaborative Filtering Algorithm

**DOI:** 10.1155/2022/1900209

**Published:** 2022-09-17

**Authors:** Yanping Du, Lizhi Peng, Shuihai Dou, Xianyang Su, Xiaona Ren

**Affiliations:** Beijing Institute of Graphic Communication, Beijing Key Laboratory of Digitalized Printing Equipment, Beijing, China

## Abstract

(*Purpose/Significance*). This paper aims at the problems of inaccurate recommendation effect caused by data sparseness and cold start in the traditional collaborative filtering-based book personalized recommendation algorithm. So this paper proposes a collaborative filtering recommendation algorithm which improves the similarity solution method and the filling method of missing data. (*Method/Process*). By considering the influence of the user's common rating book collection on the similarity calculation, the average rating value of all books is used as the threshold, and the user's common rating weight is introduced into the user's similarity calculation. As for data filling, according to the user's average rating, the basic attributes such as the age and gender of users are coded, and then Euclidean distance is initially calculated, making hierarchical clustering on users. What's more, Shope-one algorithm is used to calculate the filling value of the former *m* similar users，and add the weight value of the degree simultaneously to get the final filling value, so as to improve the data filling method. (*Result/Conclusion*). Experiments were carried out with the data set of Book-Crossing Data set through *Python*. The experimental results show that the improved collaborative filtering algorithm has a significantly improvement in the accuracy and quality of book recommendation.

## 1. Introduction

With the rapid development of cloud computing, Internet of Things, Internet and other technologies and Web 2.0, in many scenarios such as the network and digital libraries [1–3], a large amount of information data is constantly emerging from book resources, which increases people's access to the required book information difficulty, resulting in ‘information overload [4, 5]. How to solve the ‘information overload' and help users quickly find the book resources that meet their individual demand is an urgent problem to be solved [6, 7], because book recommendation algorithms can realize ‘one-to-one' service according to users' personal preferences, to provide users with personalized services book recommendations.

According to different recommendation strategies, book recommendation algorithms can be divided into: collaborative filtering-based recommendation algorithm [8], association rule-based recommendation algorithm [9], content-based recommendation algorithm [10]and hybrid recommendation algorithm [11], etc. The advantages and disadvantages of these four types of algorithms in book personalized recommendation are shown in Table 1.

Amazon [12, 13]and Dangdang [14] chose to apply the collaborative filtering-based recommendation algorithm for book recommendation services. The book recommendation based on collaborative filtering recommendation algorithm does not require in-depth analysis of the knowledge and content of book resources. It only needs to analyze the similar characteristics of users or the analysis of book borrowing records to recommend book information that users may be interested in. And the recommendation based on collaborative filtering Algorithms can handle complex structures and is technically easy to implement. Therefore, collaborative filtering recommendation algorithms are widely used. However, with the rapid increase of the number of books, the recommendation algorithm collects limited information from users in the initial stage of the system, and the traditional collaborative filtering recommendation algorithm is prone to problems such as cold start and data sparseness.

In response to the problems of data sparseness and cold start in the traditional collaborative filtering algorithm in book recommendation, domestic and foreign researchers have proposed different improvement methods. The traditional solution mainly uses the average value of the existing data to fill in the missing data, which will bring errors to the prediction results and affect the accuracy of the recommendation. In this regard, Wang [15] proposed a book recommendation algorithm based on collaborative filtering and interest, which uses the user's interest as an important measure to improve the accuracy of book recommendation. In order to effectively improve book recommendation, Zhang [16]. proposed a collaborative filtering algorithm based on time sequence, considered the student's book borrowing time and book circulation time. The experimental results showed that the accuracy of book recommendation is effectively improved. Guo [17]et al. designed a collaborative filtering recommendation algorithm based on R-RBM and Top N, which solved the problems of data sparseness, cold start and inability to mine the deep-level features of readers' personalized information in the recommendation system. Gao [18]. used the decision tree algorithm to predict the recommended books, and proposed a user-based collaborative filtering recommendation algorithm. It was confirmed by experiments that the recommendation accuracy of the algorithm is much better than the traditional collaborative filtering algorithm, which has made a big improvement. Liu [19]. used collaborative filtering algorithm for data mining of efficient book management data, and generated a book recommendation model. Noor et al. [20]. proposed a collaborative filtering method of probabilistic keywords in book recommendation system to solve the problem of data sparseness in collaborative filtering recommendation algorithm, which effectively improved the recommendation performance. Wang and Liu [21]. used association rules and collaborative filtering algorithm to mine the information of book borrowing records, and obtained the correlation of borrowing hobbies among users and the correlation between books. In summary, researcher's improved methods for the book recommendation algorithms have alleviated the problems of data sparseness and cold start in collaborative filtering recommendation algorithms for book recommendation to certain, but the amount of calculation has been increased significantly.

In view of the shortage of similarity calculation and data filling in the traditional book recommendation based on collaborative filtering algorithm, this paper improves the original data filling method by using hierarchical clustering and Slope-one algorithm to make full use of rating data, and avoids single or several user's inaccurate data filling，thus improving the filling accuracy. Then, the similarity calculation between books is added to the similarity calculation to improve the accuracy of the similarity calculation. And through the empirical analysis of the algorithm, it is verified that the improved book recommendation method based on collaborative filtering recommendation algorithm has significantly improved recommendation accuracy compared with the traditional book recommendation based on collaborative filtering recommendation algorithm.

## 2. Book Recommendation Based on Collaborative Filtering Algorithm

**Figure 1:**
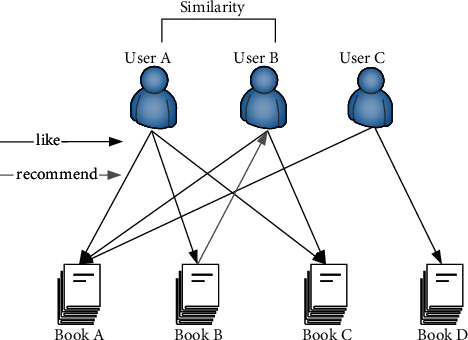
Schematic diagram of book recommendation based on user-based collaborative filtering algorithm.

The basic idea of the user-based collaborative filtering algorithm is to calculate the similarity between users according to the user's preference information and historical activity records in the system, obtain the similarity matrix between users, and find the similar neighbor set of the target user. According to the rating data of some books, it predicts the rating of target users on unrated items and selects items with high ratings for recommendation. The principle of book recommendation based on user-based collaborative filtering algorithm is shown in Figure 1.

In Figure 1, user A's preference is similar to user B's, user A prefers books A, B, and C, and user B prefers books A and C, so the algorithm can recommend book B to user B.

The traditional book recommendation based on collaborative filtering algorithm mainly includes the following three stages:

(1) Information collection: It mainly collects the user's basic information, book information, borrowing information of books and user evaluation data about books.
(2) Construction of the user rating matrix: The purpose is to reduce the time cost by converting the relevant data into dictionary format, and directly construct the query conditions according to the recommendation information when recommending, and read the relevant data. The most important part of this process is the calculation of similarity between users, that is, the similarity of users is calculated according to the books that are jointly evaluated by users, sorted by similarity, and the nearest neighbor is taken as the target and recommended to the user. The most common method for calculating the similarity of traditional collaborative filtering-based book recommendation is cosine similarity [22], Pearson similarity method [23]. In the case of high sparsity of the rating matrix, the calculation result of the Pearson similarity method is poor, and the cosine similarity is simple and opposite. Therefore, this paper uses the improved cosine similarity to calculate the similarity between users.
The cosine similarity is mainly obtained by the cosine angle between the two users, and there is an inverse relationship between the two, that is, the smaller the similarity, the larger the angle; the greater the similarity, the smaller the angle. The calculation formula of cosine similarity 1 is as follows:
  (1) $$\begin{array}{c} sim\left(a,b\right)=\cos\;\left(a,b\right)=\frac{\sum_{n\in N_{ab}}R_{a,n}R_{b,n}}{\sqrt{\sum_{n\in N_{\text{ab}}}{\left(R_{a,n}\right)}^{2}}\sqrt{\sum_{n\in N_{ab}}{\left(R_{b,n}\right)}^{2}}}\text{.} \end{array}$$
In formula (1), a, *b* represent respectively user a and user *b*, *R*_a,n_ is user b's rated for book *n*, *N*_ab_ is the set of common rating for books by users a and b,*N*_a_ is the collection of book rating for user a，*N*_b_ is the collection of book rating for user *b*.
(3) Book recommendation list generation: After determining the target neighbor users according to the similarity, it is necessary to predict the score of the target book by the neighbor users to generate the book recommendation result of the target user, and realize the personalized service of book recommendation for the user. Commonly used predictive scoring methods include average scoring method [24] and offset weighted scoring method [25].

**Figure 2:**
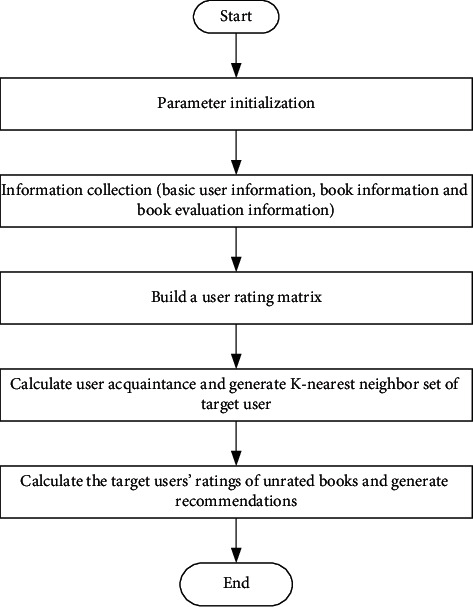
Traditional book recommendation process based on collaborative filtering algorithm.

The traditional book recommendation process based on collaborative filtering algorithm is shown in Figure 2.

## 3. Improvement of Book Recommendation Based on Collaborative Filtering Algorithm

The traditional book recommendation based on collaborative filtering algorithm only calculates the similarity between users according to the user's basic information and the user's rating of the book, and then recommends the books with high ratings to the user. With the exponential growth of users and the number of books in the book recommendation system, the user's rating data will be very sparse, which will have a great impact on the accuracy of similarity calculation, resulting in a larger error in the accuracy of book recommendation results, which affects the recommendation quality. According to the problems of data sparsity and cold start in book recommendation based on traditional collaborative filtering algorithm, this paper proposes improvements in similarity calculation and data filling.

### 3.1. Improvement of Similarity Calculation Method

Similarity calculation is the key to finding the nearest neighbor set. The traditional user similarity calculation is to directly calculate the similarity between different users based on whether the target user has the same interest and hobbies for a certain book, different historical scoring data and so on. In this paper, aiming at the problem that there are very few common scores of books between two neighboring users in the traditional similarity calculation method, this paper introduces the user's common scoring weight factor to improve the accuracy of the cosine similarity result. User common rating weight is defined as:

(2) $$\begin{array}{c} {\bar{N}}_{\text{ab}}=\overset{n}{\underset{a,b=0}{\sum }}\frac{N_{a}+N_{b}}{2}\text{.} \end{array}$$

The improved user similarity calculation method is:

(3) $$\begin{array}{c} {\text{COSU}}_{a,b}=\text{norm}\left(N_{a}\right)*\text{norm}\left(N_{b}\right)*\text{norm}\left(N_{\text{ab}}\right)\text{,} \end{array}$$

(4) $$\begin{array}{c} S\text{im}U_{a,b}=0.5+0.5*\text{COS}U_{a,b}\text{.} \end{array}$$

In formulas 2, 3, 4, *S*im*U*_a,b_ represents the similarity between users a and b，${\bar{N}}_{\text{ab}}$ is the average score of users a and *b* for the jointly rated books.

After the improvement and fully consideration, the influence of the common scores between users on the similarity calculation can effectively improve the similarity calculation between users.

### 3.2. Improvements of Data Filling

The historical users in book recommendation can not score all books, and there will be a large amount of null data between the constructed user-book model, which will directly affect the accuracy of user similarity calculation, resulting in a plummeting recommendation quality. To face this problem, it is necessary to pre-process the data, filter and delete some user data without a large number of scores, and fill in the missing part of the data with specific data to ensure data integrity. The general data filling method does not significantly improve the final result. In order to effectively improve the quality of user recommendation, this paper uses the hierarchical clustering algorithm and the improved Slope-one algorithm to improve the data filling method.

In order to improve the efficiency of users' data analysis of book-related information and reduce the sparsity of the score matrix, a hierarchical clustering of users is established according to the user's historical record information on books [26, 27], and users are divided into high-rating (*U*_o_) and low-rating (*U*_p_) and medium rating (*U*_n_) three user groups to achieve dimensionality reduction processing of data and reduce the amount of calculation. That is, if the average user's rating for books is above 4, it is *U*_o_, then if the average user's rating for books is less than 2, it is *U*_p_, and finally, if the average user's rating for books is between 2 and 4, it is *U*_n_.

The clustering process is as follows:

(5) $$\begin{array}{c} If\bar{r_{n,i}}>=4r_{n}\in U_{o}\text{,}\\ ElseIf\bar{r_{n,i}}<=2r_{n}\in U_{p}\text{,}\\ Else2<=\bar{r_{n,i}}<=4r_{n}\in U_{n}\text{.} \end{array}$$

Where $\bar{r_{n,i}}$ is the average rated of book *n* by user *i*.

Through the process of hierarchical clustering, each group of data after clustering is filled with missing data to reduce the dimension of the data and reduce the amount of calculation. The similarity between users in the same group needs to be considered before filling. The traditional data filling method only uses the historical scoring data to solve the Euclidean distance as the similarity value, but the newly added users have no historical scoring information and cannot calculate the Euclidean distance. Therefore, this paper introduces user attributes，which is added to the similarity calculation.

The user information is encoded using One-hot coding, that is, the user's score for each book, with 0 means no score or a rating of 0. The encoded user information is obtained, and the similarity value is obtained. Using the Slope-one algorithm to calculate the filling data for the missing values of the first *m* users, and adding the weight of the similarity, the filling value is finally obtained. The selection of the padding value needs to be within a suitable range. If the selected value is too large or too small, the filling accuracy will be affected.

The basic idea of the Slope-one algorithm is a linear algorithm that calculates the difference between the user's scores on different books, and predicts the score of another book according to the user's score on a certain book. The specific calculation formula is as follows:

(6) $$\begin{array}{c} \bar{R\left(a,b\right)}=\frac{\sum_{i\in N_{\text{ab}}}\left(r_{\text{ai}}-r_{\text{bi}}\right)}{\text{card}\left(N_{\text{ab}}\right)}, \end{array}$$

(7) $$\begin{array}{c} T_{\text{bj}}=r_{\text{aj}}-\bar{R\left(a,b\right)}\text{.}, \end{array}$$

In formulas (5), (6), $\bar{R\left(a,b\right)}$ represents the average score difference between users a and *b* for each jointly rated book;*r*_ai_ is the rating value of user a for book *i*, *r*_bj_is the rating value of user *b* for book i;card(*N*_ab_)is the total number of books rated both a and b,*P*_bj_is the filling data information of user b's rating of book j,*r*_aj_is the filling data information of user a's rating of book *j*.

The formula for filling the value of each missing value:

(8) $$\begin{array}{c} D\left(u,v\right)=\sqrt{\underset{i\in I_{u,v}}{\sum }{\left(r_{u,i}-r_{v,i}\right)}^{2},} \end{array}$$

(9) $$\begin{array}{c} Sim_{a,b}=D\left(u,v\right)\times a_{u,v}\text{,} \end{array}$$

(10) $$\begin{array}{c} T_{bj}=r_{aj}-\frac{\underset{i\in N_{\text{ab}}}{\sum }\left(r_{\text{ai}}{-r}_{\text{bi}}\right)}{\text{card}\left(N_{\text{ab}}\right)}\text{,} \end{array}$$

(11) $$\begin{array}{c} T_{wj}=\frac{\sum_{u=1}^{n}\left(S{\text{im}}_{u,w}\times T_{u,j}\right)}{\sum_{u=1}^{n}S{\text{im}}_{u,w}}\text{.} \end{array}$$

In formulas (5), (6),*D*(*u*, *v*) is the encoded Euclidean distance,*r*_*u*,*i*_is the vector composed of the user's information code and the score for book i,*a*_*u*,*v*_ is the proportion of items scored together,*S*im_*a*,*b*_ is the similarity between the user and the added user's specific information and scoring together,*T*_*u*,*j*_is the specific padding value for user *u* and book *j* in terms of missing data, and *T*_w*j*_ is the final padding value.

Data filling process:

**Step 1 .**

According to the basic attributes of users in the data set, perform hierarchical clustering on users, and divide all users into three different user categories.

**Step 2 .**

According to different user categories, use formula (9) for the user groups to calculate the similarity in the same group, and find the neighbor user set;

**Step 3 .**

Use formula (10) to fill in the missing values in the system;

**Step 4 .**

Introduce the similarity calculation weight to optimize the filling data;

**Step 5 .**

Repeat the above steps until the entire user data set is populated.

In summary, hierarchical clustering is performed on users according to user ratings, which achieves the effect of initial dimensionality reduction, and at the same time, the accuracy of similarity calculation after clustering is improved to a certain extent. The basic information of the user is added when calculating the similarity, which effectively alleviates the cold start problem. The accuracy of the similarity is further improved by adding the weight of the common rating.

### 3.3. Prediction Score

The set of neighbor users of the target user is obtained by similarity calculation as *U*={u_1_, u_2_, u_3_, ⋯, u_n_}, according to the score of each neighbor user on the target book, the filling formulas (10) and (11) are used to predict the score of the target book [28, 29]. The prediction result of the i-th target user is, and then use the prediction formula (12) to predict and score the final target book, complete the prediction of all target book scores, and form the final book recommendation result.

According to the calculation of similarity, the sore prediction formula is as follows:

(12) $$\begin{array}{c} r\left(u,g\right)=\bar{r_{u}}+\frac{\sum_{k\in \bar{U}}\text{sim}\left(u,k\right)\left(r_{k,i}-\bar{r_{k}}\right)}{\sum_{k\in \bar{U}}\left|sim\left(u,k\right)\right|}\text{.} \end{array}$$

In formula (12),$\bar{r_{u}}$ is the average rating of all books by user u,$\bar{r_{k}}$is the average rating of all books by neighbor user *k*.

### 3.4. Improved Book Recommendation Process

The improvement of the collaborative filtering algorithm is mainly divided into three steps: Firstly, for the improvement of data filling method, build a user-book model, perform hierarchical clustering on users, encode the basic information of users and initially calculate the Euclidean distance, and the introduce of Shope-one algorithm to fill in the missing data; secondly, the improvement of similarity calculation method, considering the influence of the common score of neighboring users on the similarity calculation, an improved similarity calculation method is introduced to calculate the similarity of neighboring users; finally, the improved collaborative filtering algorithm is used to predict users' ratings of unselected books for recommend books to users.

**Figure 3:**
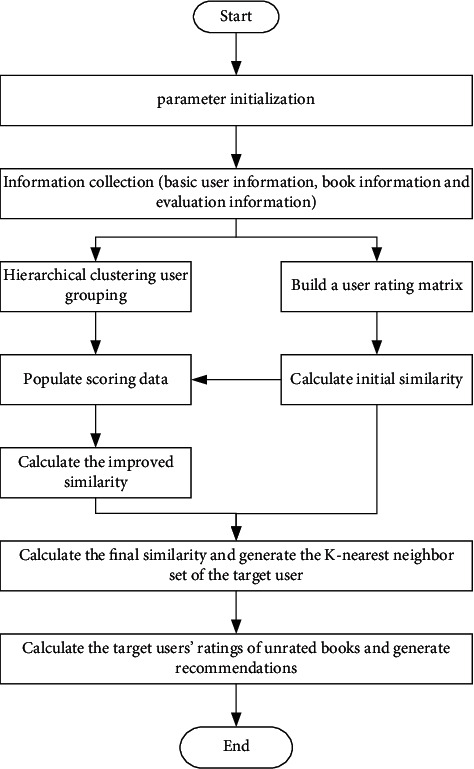
Workflow of the improved algorithm in the book personalized book recommendation system.

The book recommendation process based on the improved collaborative filtering recommendation is shown in Figure 3.

## 4. Analysis of Experimental Results

Firstly, the experimental environment, the selected data set and the experimental evaluation indicators involved in this paper are briefly introduced, and then the method proposed in this paper and the method before improvement are compared and analyzed according to the data set.

### 4.1. Introduction to Experimental Environment and Data Set

The computer configuration used in this study is Intel Core i5-6200 CPU with 4 GB of running memory. The operating system is Windows 7 64 bit, the programming language is *Python* language, the version is *Python* 3.8, and the editor is Anaconda Jupyter Notebook.

The data used in this article comes from the Book-Crossing Data set released by the Free University of Germany. The Book-Crossing Data set is composed of the scores of 278,858 users in the Book-Crossing community, including 1,149,780 book scoring data of about 271,379 books, with a score of 1∼ 5 points, the Data set contains 3 categories: BX-users, BX-books, and BX-book ratings. In this experiment, on the one hand, the experimental results are obtained from the MASE value of the improved similarity calculation and data filling; on the other hand, the final experimental results are obtained by calculating the precision, recall and F-measure of the book recommendation results.

### 4.2. Algorithm Evaluation Index and Experimental Results

#### 4.2.1. Predictive Scoring Accuracy of the Algorithm

*(1) Experimental Evaluation Index*. The performance evaluation indexes of the recommended algorithms mainly include mean square error (MSE), root mean square error (RMSE), square absolute error (MAE), precision, recall and so on. The accuracy of the predicted score refers to the difference between the predicted score of the recommendation algorithm and the actual score of the user. This paper uses RMSE as the evaluation index. The smaller the RMSE value, the better the accuracy of the prediction results.

Assuming that the set of predicted scores for the book by the recommendation system is{p_1_, p_2_, p_3_, ⋯, p_n_}, and the actual score of the book is{q_1_, q_2_, q_3_, ⋯, q_n_}, then the RMSE can be expressed as formula (12).

(13) $$\begin{array}{c} RMSE=\sqrt{\frac{{\sum_{i=1}^{n}\left(p_{i}-q_{i}\right)}^{2}}{N}}\text{.} \end{array}$$

In formula (12), p_i_ is the predicted rating, q_i_ is the actual rating, and N is the total number of predicted books.

*(2) Experimental Results and Analysis*. Method 1: Selected different number of neighbor user, which is 5, 10, 15, 20, 25, 30, 35, 40, 45, and 50 to analyze the experimental results. In the experiment, users in the data set were randomly selected for prediction and scoring experiments, and the method of similarity calculation and data filling before and after the improvement of personalized book recommendation based on collaborative filtering algorithm was adopted. The results are shown in Table 2.

**Figure 4:**
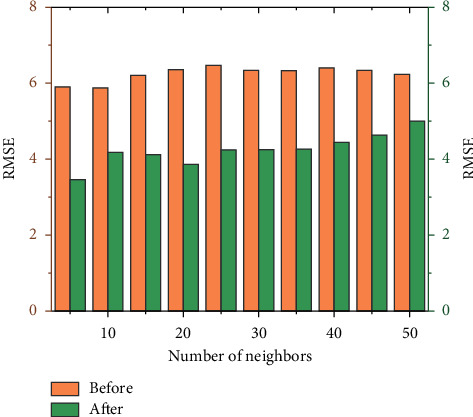
RMSE values of nearest neighbor users with different number of clusters.

Figure 4 shows the change of RMSE value. The abscissa represents the number of neighboring users with different numbers of clusters, and the ordinate represents the RMSE value of the predicted scoring result. It can be seen from the figure that the RMSE value of the improved algorithm is always lower than that of the traditional algorithm, and the RMSE value of the improved algorithm is reduced by an average of 32% compared with the traditional algorithm. Because the smaller the RMSE value, the better the recommendation effect of the algorithm is. The proposed improvements in similarity calculation and data filling are better than the unimproved methods, and the improved recommendation algorithm is better than the traditional recommendation algorithm in the cold-start environment.

Method 2: Method 1 mainly conducts experiments from neighboring users with different cluster numbers. In order to further verify that the similarity calculation and data filling method proposed in this paper is better than the unimproved method, we select 100 neighbors with the same number of clusters and different neighbors. The user conducts multiple experimental results analysis, and the experimental results are shown in Table 3.

**Figure 5:**
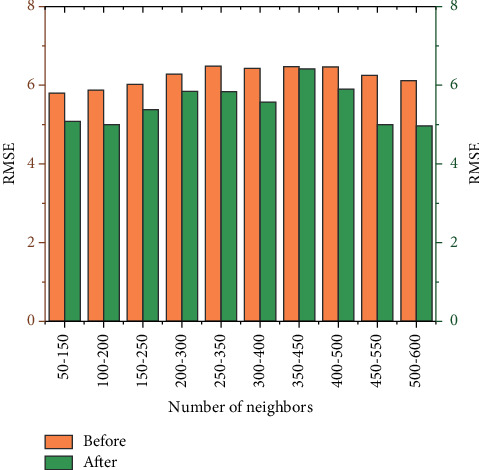
RMSE values of users with the same number of clusters but different neighbors.

Figure 5 shows the change of RMSE value of 100 neighbors with the same number of clusters selected each time. The abscissa represents the 100 neighbors in different intervals in the data set, and the ordinate represents the RMSE value of the predicted scoring result. It can be seen from the figure that the RMSE value of the improved algorithm is always lower than that of the traditional algorithm, and the RMSE value of the improved algorithm is reduced by an average of 12% compared with the traditional algorithm. Because of the smaller the RMSE value, the better the recommendation effect of the algorithm is. The proposed similarity computation improvements and data padding improvements are compared to the unimproved methods, and the results are consistently better than the unimproved traditional methods.

#### 4.2.2. The Accuracy of the Recommended Results

*(1) Experimental Evaluation Index*. Based on the results of using different numbers of neighbor users from 5 to 50, and the results of selecting 100 different neighbor users in different intervals each time, the RSME prediction error of the improved algorithm is smaller than that of the traditional algorithm. In order to evaluate the correlation prediction accuracy of the recommendation results in this algorithm, this paper selects three evaluation indicators related to the effect of book recommendation, namely accuracy, recall, and F-measure. These three evaluation indicators are used to compare book recommendation based on traditional collaborative filtering algorithm with the improved collaborative filtering algorithm-based book recommendation is compared.

The accuracy rate indicates the accuracy of the recommendation in the recommendation list, that is, the proportion of the total number of books that are successfully recommended. The recall rate indicates the proportion of the books that users recommend accurately in the selected experimental data set, that is, the proportion of successfully recommended books to the user's interest. Among them, the calculation formula of precision rate (14) is shown, and the formula of recall rate (15).

(14) $$\begin{array}{c} Precision=\frac{\sum_{u\in U}\left|R_{u}\cap I_{u}\right|}{\sum_{u\in U}\left|R_{u}\right|}\text{,} \end{array}$$

(15) $$\begin{array}{c} Re\text{call}=\frac{\sum_{u\in U}\left|R_{u}\cap I_{u}\right|}{\sum_{u\in U}\left|I_{u}\right|}\text{.} \end{array}$$

In formulas (13) and (14), *R*_u_ is the target user *U*_i_ recommends *R* books, and *I*_u_ is the collection of books that he likes during the experiment.

*F*-measure is calculated by the combination of precision and recall. When there is a contradiction between precision and recall, F-measure is usually used to evaluate the efficiency of the recommendation algorithm. The higher the F-measure value, the higher the effectiveness of the algorithm. The *F*-measure calculation formula is shown in (3)-(4).

(16) $$\begin{array}{c} F=\frac{2\times recall\times precision}{recall+precision}\text{.} \end{array}$$

*(2) Experimental Results and Analysis*. Method 1: For the book personalized recommendation results based on collaborative filtering algorithm, the main influence is the number of clustered nearest neighbor users. In the experiment, the values of nearest neighbor users are selected as 5, 10, 15, 20, 25, 30, 35, 40, 45 and 50 respectively to analyze the experimental results.

**Figure 6:**
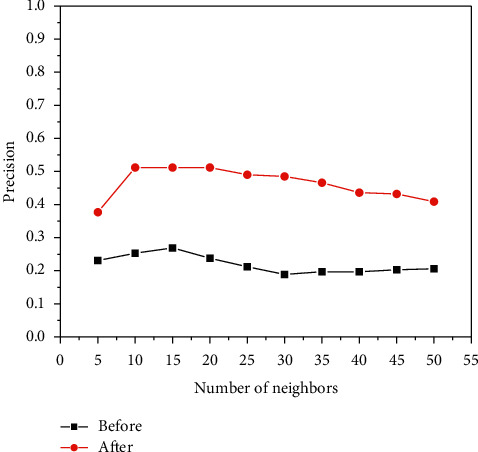
The precision of neighbor users with different numbers of clusters.

**Figure 7:**
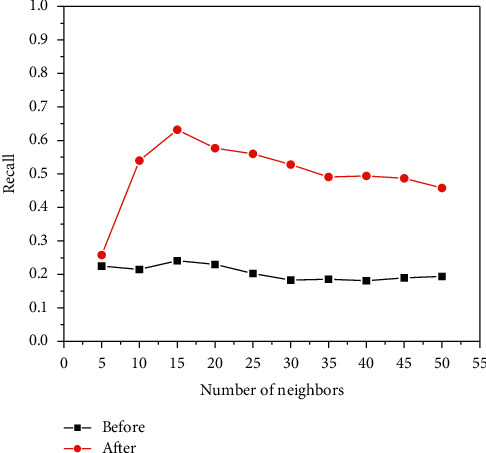
The recall rate of neighbor users with different numbers of clusters.

**Figure 8:**
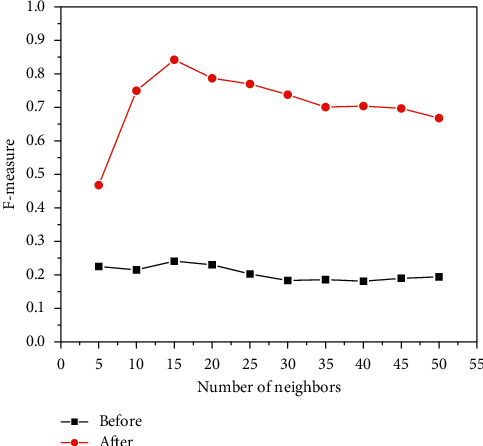
F-measure values of neighbor users with different numbers of clusters.

Figure 6, Figure 7, and Figure 8 show the experiments of the precision, recall, and *F*-measure of the improved book recommendation based on collaborative filtering algorithm and the traditional book recommendation based on collaborative filtering algorithm. As a result, the abscissas all represent the number of neighboring users with different numbers of clusters, the ordinates in Figure 6 represent the precision of the predicted scoring results, the ordinates in Figure 7 represent the recall rates of the predicted scoring results, and the ordinates in Figure 8 The ordinate represents the *F*-measure value of the predicted scoring result. As can be seen in Figure 6, Figure 7, and Figure 8, the precision, recall, and *F*-measure of the traditional book recommendation results are always lower than those of the improved book recommendation results. rate, *F*-measure value. When the number of clusters reaches 15, the precision, recall, and *F*-measure value all reach an optimal value, indicating that the recommendation effect is the best at this time. It can be seen that the precision, recall, and *F*-measure have experienced a process of first increase and then decrease with the increase of the number of clusters of neighboring users, which conforms to the general law of precision, recall, and *F*-measure value, indicating that this paper The proposed improvements to the collaborative filtering-based recommendation algorithm can effectively improve the quality of book recommendation.

**Figure 9:**
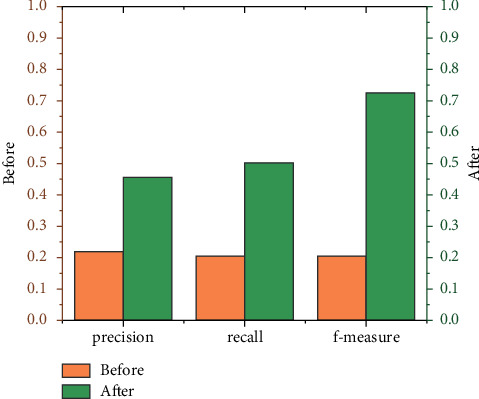
Average values of various index of neighboring users with different numbers of clusters.

Figure 9 is a comparison chart of the precision, recall, and average value of *F*-measure of each index of neighboring users with different numbers of clusters. The average precision rate is about 52% higher than that of traditional book recommendation, and the average recall rate is higher than the traditional book recommendation，which is improved by about 59%, and the average *F*-measure value is about 72% higher than that of the traditional book recommendation. It can be seen that the improved algorithm in this paper has achieved a very significant improvement in the recommendation effect. The accuracy and intelligence of the recommendation provide support for users to provide more personalized information services.

Method 2: Method 1 is mainly to verify the neighboring users with different numbers of clusters to conduct experiments. In order to further verify that the improved personalized book recommendation based on the collaborative filtering recommendation algorithm is better than the unimproved algorithm, in the experiment, 100 users are selected each time. The experimental results are analyzed for users with the same number of clusters and different neighbors.

**Figure 10:**
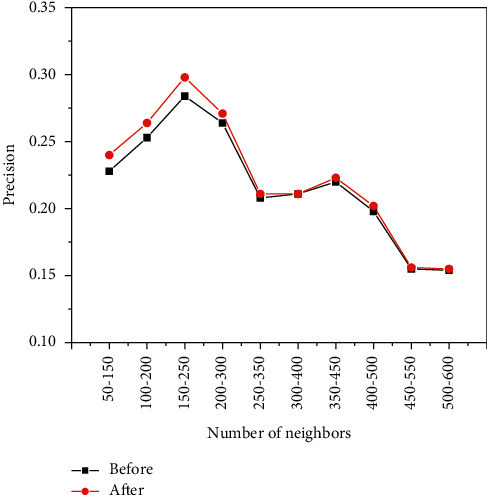
The precision of users with the same number of clusters but different neighbors.

**Figure 11:**
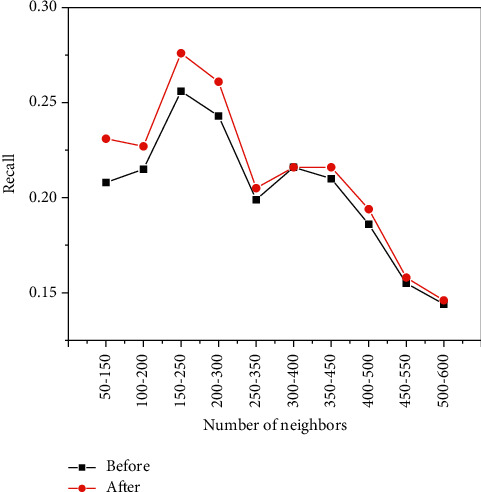
The recall of users with the same number of clusters and different neighbors.

**Figure 12:**
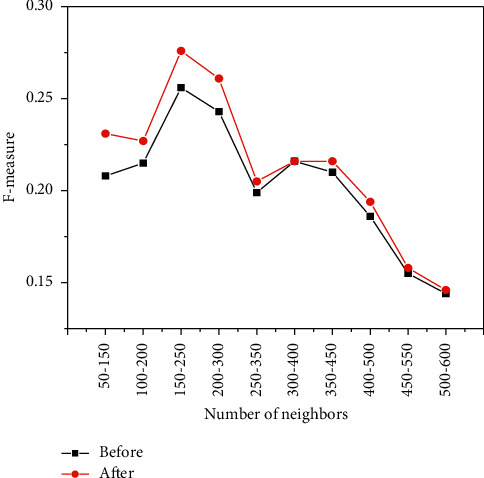
The F-measure values of users with the same number of clusters and different neighbors.

Figure 10, Figure 11, Figure 12 show the experiments of the precision, recall, and *F*-measure of the improved book recommendation based on collaborative filtering algorithm and the traditional book recommendation based on collaborative filtering algorithm. As a result, the abscissas all represent 100 neighbors with the same number of clusters in different intervals in the data set, the ordinates in Figure 10 represent the precision of the predicted scoring results, and the ordinates in Figure 11 represent the recall of the predicted scoring results. rate, and the ordinate in Figure 12 represents the *F*-measure value of the predicted scoring result. As can be seen in Figure 10, Figure 11, and Figure 12, the accuracy, recall, and *F*-measure of traditional book recommendation results for 100 neighboring users in different intervals are always lower than those of the improved books' precision, recall, and *F*-measure value of the recommended results are in line with the general rules of precision, recall, and *F*-measure value, which further shows that the improvement of the recommendation algorithm based on collaborative filtering proposed in this paper can effectively improve the quality of book recommendation.

**Figure 13:**
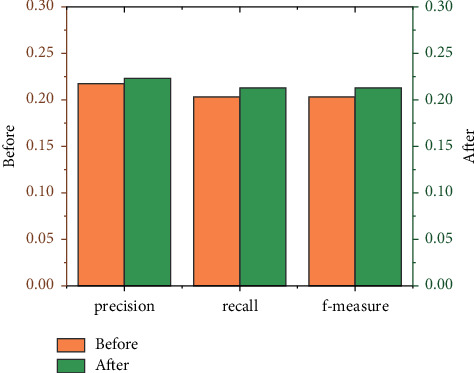
Average values of each index for users with the same number of clusters and different neighbors.

Figure 13 is a comparison chart of the precision, recall, and average value of *F*-measure for the same number of clusters and different neighbors. It can be seen that the improved algorithm in this paper has 100 users with the same number of clusters in different intervals. The improvement of the recommendation results above further shows that the improved results of the book recommendation based on the collaborative filtering algorithm proposed in this paper are better than the traditional book recommendation based on the collaborative filtering algorithm.

## 5. Conclusion

In order to improve the accuracy of book recommendation and predict the needs of users' books, the traditional book recommendation based on collaborative filtering algorithm has data sparse and cold-start problems in similarity calculation and data filling. In this paper, the slop-one algorithm is used to improve the way of data filling, the common score of neighboring users is introduced to improve the similarity calculation. And a book recommendation based on the improved collaborative filtering algorithm is proposed. The basic information, book information and evaluation information are hierarchically clustered, and the missing data values are filled, and then the similarity calculation is performed, which can easily solve the problems of data sparseness and cold start encountered by traditional algorithms. Through the verification of the algorithm improvement based on *Python*, the experimental results show that under the sparse data, compared with the traditional book recommendation based on collaborative filtering algorithm, the improved book recommendation based on collaborative filtering algorithm has smaller prediction error, precision, recall, *F* -measure value is higher. Therefore, the improved book recommendation based on collaborative filtering algorithm solves the problems of data sparseness and cold start, and helps to provide users with high-quality book recommendations and realizes personalized services. However, the improved algorithm in this paper also has shortcomings. On the one hand, with the passage of time, users' interest in books may change, and historical data is time-sensitive, which may result in poor book recommendation results. Compared with the previous improvement, book recommendation consumes a longer time, and which will be important research directions for the next step.

## Figures and Tables

**Table 1:** Comparison of advantages and disadvantages of book recommendation algorithms.

Book recommendation algorithm	Advantage	Disadvantages
Content-based recommendation algorithms	Easy to implement, simple method, high recommendation quality, and real-time	The algorithm for extracting content is complex and difficult to process
Recommendation algorithm based on association rules	High recommendation quality	Difficulty in data mining, low computational efficiency, poor personalized recommendation
Collaborative filtering recommendation algorithms	Simple method and can handle complex structures, and better personalized recommendation	Cold start problems, data sparse problems
Hybrid recommendation algorithm	Comprehensive use, high recommended quality	The algorithm is complex and the calculation is difficult

**Table 2:** RMSE values of neighbor users with different numbers of clusters before and after algorithm improvement.

Number of clustered users	RMSE value of traditional collaborative filtering recommendation algorithm	RMSE value of improved collaborative filtering recommendation algorithm
5	5.903	3.469
10	5.878	4.184
15	6207	4.123
20	6.355	3.86
25	6.47	4.243
30	6.339	4.252
35	6.333	4.263
40	6.408	4.444
45	6.34	4.637
50	6.234	5

**Table 3:** RMSE values of users with the same number of clusters and different neighbors before and after the improvement of the algorithm.

Number of clustered users	RMSE value of traditional collaborative filtering recommendation algorithm	RMSE value of improved collaborative filtering recommendation algorithm
100	5.801	5.082
100	5.878	5
100	6.02	5.378
100	6.282	5.846
100	6.483	5.841
100	6.426	5.568
100	6.474	6.414
100	6.466	5.898
100	6.252	5
100	6.119	4.966
Average	6.2201	5.4993

## Data Availability

The data for all Figures used to support the findings of this study are included within the article.
